# Deoxyguanosine is a TLR7 agonist

**DOI:** 10.1002/eji.201948151

**Published:** 2019-11-14

**Authors:** Tamara Davenne, Anne Bridgeman, Rachel E. Rigby, Jan Rehwinkel

**Affiliations:** ^1^ Medical Research Council Human Immunology Unit, Medical Research Council Weatherall Institute of Molecular Medicine, Radcliffe Department of Medicine University of Oxford Oxford UK; ^2^ Laboratory for Disease Mechanisms in Cancer Department of Oncology KU Leuven and Leuven Cancer Institute (LKI) Leuven Belgium

**Keywords:** deoxyguanosine, guanosine, R848, ssRNA, TLR7

## Abstract

Toll‐like receptor 7 (TLR7) is an innate immune sensor for single‐strand RNA (ssRNA). Recent structural analysis revealed that TLR7 has an additional binding site for nucleosides such as guanosine, and is activated when both guanosine and ssRNA bind. The nucleoside binding site also accommodates imidazoquinoline derivatives such as R848, which activate TLR7 in the absence of ssRNA. Here, we report that deoxyguanosine (dG) triggered cytokine production in murine bone marrow derived macrophages and plasmacytoid dendritic cells, as well as in human peripheral blood mononuclear cells, including type I interferons and pro‐inflammatory factors such as TNF and IL‐6. This signalling activity of dG was dependent on TLR7 and its adaptor MyD88 and did not require amplification via the type I interferon receptor. dG‐triggered cytokine production required endosomal maturation but did not depend on the concurrent provision of RNA. We conclude that dG induces an inflammatory response through TLR7 and propose that dG is an RNA‐independent TLR7 agonist.

## Graphical abstract

Toll‐like receptor 7 (TLR7) is a pattern recognition receptor and plays important roles in virus infection and autoimmunity. TLR7 detects single‐stranded RNAs, nucleosides, and imidazoquinoline derivatives. Davenne et al. show that deoxyguanosine activates an inflammatory gene expression programme by engaging TLR7 and propose that this activity does not require RNA binding to TLR7.

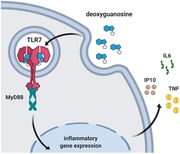

## Introduction

PRRs are germline encoded proteins that recognise PAMPs and/or danger‐associated molecular patterns. TLRs are a class of PRRs. Their ligand binding domains survey the extracellular environment or endosomal lumen and consist of multiple leucine‐rich repeats forming horseshoe‐shaped structures 1. A single transmembrane helix connects to a C‐terminal cytoplasmic Toll/interleukin‐1 receptor homology (TIR) domain that is required for signalling. Upon binding to agonists, TLRs form active dimers and TIR domains recruit the adaptor molecules myeloid differentiation primary response 88 (MyD88) or TIR domain‐containing adaptor inducing interferon β (TRIF) 2. Downstream signalling then leads to the induction of an innate immune response, a hallmark of which is the secretion of cytokines.

The TLR7 subfamily includes the endosomal receptors TLR7, TLR8, and TLR9, which all sense nucleic acids and signal via MyD88 2. Although TLR7 was first reported to be activated by imidazoquinoline compounds and guanosine analogues 3, 4, 5, 6, it is best known as a sensor of single strand RNAs (ssRNAs) of viral or self‐origin and recognises uridine ribonucleotides in ssRNA 7, 8, 9. Structural analysis of TLR7 revealed the presence of two binding sites in its extracellular domain 10. The first binding site can be occupied by endogenous nucleosides, nucleoside analogues, or imidazoquinoline compounds, whereas the second binding site recognises ssRNA 10, 11. Interestingly, the imidazoquinoline R848 dimerises TLR7 in the absence of ssRNA, while TLR7 activation by natural nucleosides requires ssRNA binding to the second binding site 10, 11, 12. Indeed, guanosine, deoxyguanosine (dG), and their derivatives 8‐hydroxyguanosine (8‐OHG) and 8‐hydroxydeoxyguanosine (8‐OHdG) have been reported to enhance TLR7 signalling triggered by ssRNA but not to signal on their own in WT cells 12.

A more comprehensive understanding of how nucleosides and ssRNAs activate TLR7 may facilitate the design of TLR7 specific agonists and antagonists. TLR7 agonists can be used as vaccine adjuvants or to boost anti‐tumour immunity, while antagonists may be beneficial in conditions such as systemic‐lupus erythematosus or rheumatoid arthritis, where TLR7 mediates the production of type I IFN. Here, we show that TLR7 recognises dG, resulting in a pro‐inflammatory response. Surprisingly, dG triggered TLR7 in the absence of ssRNA stimulation. Like imidazoquinoline compounds, dG may thus be a ssRNA‐independent agonist for TLR7.

## Results and discussion

### dG induces cytokine secretion in macrophages

**Figure 1:**
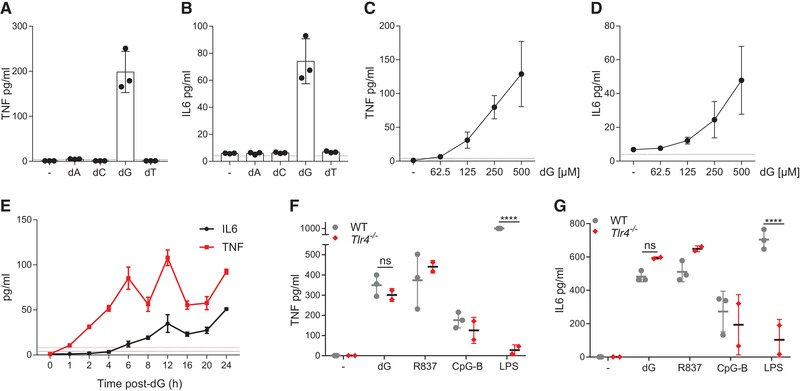
BMDMs secrete cytokines upon dG treatment. (A, B) BMDMs were treated with 0.5 mM of the indicated dNs for 24 h. TNF (A) and IL6 (B) concentrations in supernatants were determined by ELISA. (C, D) The experiment in (A,B) was repeated with the indicated concentrations of dG. (E) BMDMs were treated with 0.5 mM dG for the indicated periods of time and supernatants were analysed as in (A, B). (F, G) BMDMs of the indicated genotypes were treated with 0.5 mM dG, 2.5 µg/mL R837, 2.5 µg/mL CpG‐B DNA, or 2.5 ng/mL of LPS for 24 hours. Supernatants were analysed as in (A, B). Pooled data from biological replicates (BMDM cultures originating from individual mice, *n* = 3 for WT and *n* = 2 for *Tlr4^−/−^*) are shown with mean ± SD. Data are representative of three (A, C) or two (B, D–G) independent experiments. Dashed lines represent detection limits. *p*‐values determined with two‐way ANOVA are indicated. ns, *p* ≥0.05; ****, *p* < 0.0001.

To study the role of deoxyribonucleosides (dNs) in TLR7 activation, we treated murine bone‐marrow derived macrophages (BMDMs) with dNs and analysed the production of proinflammatory cytokines. Treatment of BMDMs with dG induced the secretion of TNF and IL‐6 (Fig. 1A and B). In contrast, deoxyadenosine, deoxycytidine, and deoxythymidine did not trigger the production of detectable levels of these cytokines (Fig. 1A and B). We therefore used dG in subsequent experiments. Titration of dG revealed a dose‐dependent induction of TNF and IL‐6, starting at ∼100 µM dG (Fig. 1C and D). A time‐course experiment showed that TNF was secreted as early as 2 h post‐dG treatment, followed by IL‐6, which was detectable from 6 h onwards (Fig. 1E).

BMDMs produce cytokines upon sensing of endotoxins, such as LPS, sometimes present as contaminants in reagents. LPS from gram negative bacteria is sensed by TLR4 2. To demonstrate the absence of endotoxin contamination, we used *Tlr4^−/−^* BMDMs. As expected, *Tlr4^−/−^* cells did not secrete TNF and IL6 in response to LPS stimulation, while the response to the TLR7 and TLR9 agonists R837 and CpG‐B, respectively, was unchanged (Fig. 1F and G). Importantly, dG induced comparable amounts of TNF and IL6 in WT and *Tlr4^−/−^* BMDMs (Fig. 1F and G). Therefore, cytokine secretion induced by dG was not due to the presence of contaminating endotoxins. We further confirmed this conclusion by polymyxin B treatment of dG and LAL test (data not shown). These observations suggest the presence of a dG sensor.

### dG is detected by TLR7 and MyD88

**Figure 2:**
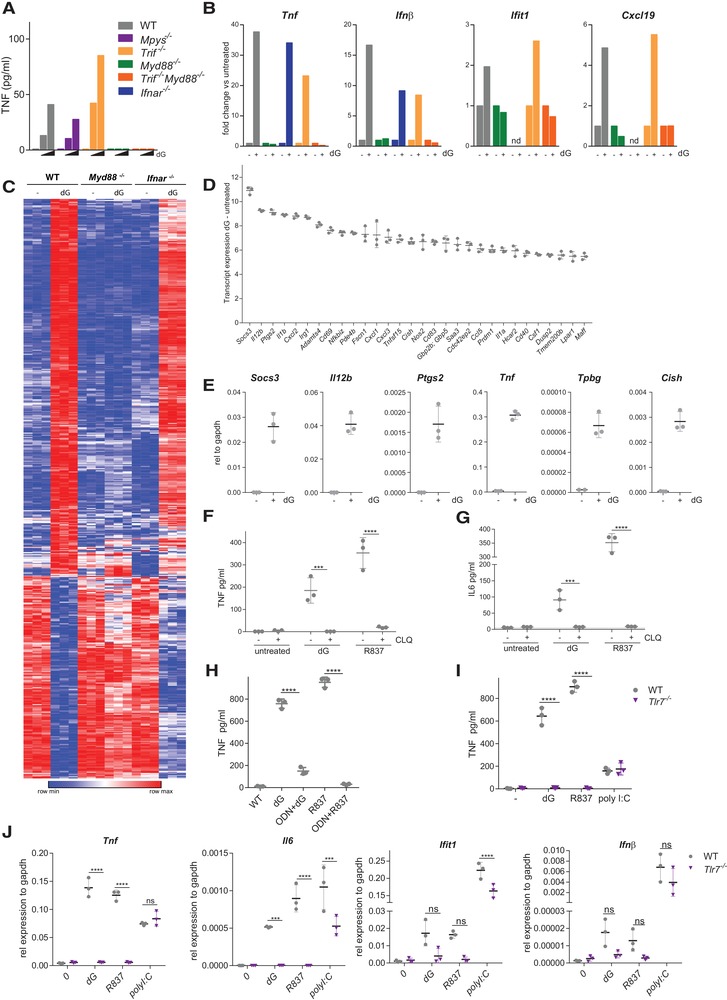
dG triggers a pro‐inflammatory response through MyD88 and TLR7. (A, B) BMDMs of the indicated genotypes were treated with 0.5 mM dG for 24 h. (A) Supernatant TNF concentrations were determined as in Fig. 1A. (B) Expression of the indicated mRNAs was analysed by RT‐qPCR. Data are relative to *Gapdh* and were normalised to untreated cells. (C–E) BMDMs prepared from three mice per genotype were treated with 0.5 mM dG for 3 hours or left untreated. RNA was extracted for microarray (C, D) and RT‐qPCR analysis (E). (C) 538 transcripts more than 2‐fold up‐ or down‐regulated after dG treatment in WT cells (FDR < 0.05) are shown in rows after hierarchical clustering. Each column represents BMDMs from one animal. Colours represent minimum and maximum expression per transcript. (D) The 30 most upregulated genes from (C) are shown for WT cells. (E) Expression of selected transcripts was validated by RT‐qPCR in WT cells. Data are relative to *Gapdh*. (F, G) BMDMs were treated with 10 µg/mL chloroquine (CLQ) and 0.5 mM dG or 200 ng/mL R837 as indicated for 24 h. Supernatants were analysed as in Fig. 1A and B. (H) BMDMs were pre‐treated or not with 2 µM ODN20958 for 1 h. Cells were then treated for 24 h with 0.5 mM dG or 200 ng/mL R837. Supernatants were analysed as in Fig. 1A. (I, J) BMDMs of the indicated genotypes were treated for 24 h (I) or 6 h (J) with 0.5 mM dG, 200 ng/mL R837 or 200 ng/mL poly I:C. (I) Supernatant TNF concentrations were determined as in Fig. 1A. (J) Expression of the indicated mRNAs was analysed as in (E). Data in (A, B) are averages from technical duplicates and are representative of two independent experiments. In (C–J) pooled data from biological replicates (*n* = 3) are shown with mean ± SD and are representative of three (I, J), two (F–H), or one (C–E) independent experiments. *p*‐values determined with two‐way ANOVA are indicated. ns, *p* ≥0.05; ***, *p* < 0.001; ****, *p* < 0.0001.

To determine whether dG was sensed by a TLR, dG treatment was performed in BMDMs lacking the adaptor protein TRIF, which acts downstream of TLR3 and TLR4, and/or MyD88, which acts downstream of all TLRs except TLR3 2. The adaptor protein STING mediates signalling of the cytosolic DNA sensor cGAS, and *Mpys^−/−^* BMDMs (lacking STING) were used as an additional control. Like WT cells, *Mpys^−/−^* and *Trif^−/−^* BMDMs produced TNF upon dG treatment (Fig. 2A). In contrast, *Myd88^−/−^* and *Trif^−/−^; MyD88^−/−^* BMDMs did not secrete TNF. To further substantiate this result, we quantified mRNA levels by RT‐qPCR. *Tnf* and *Ifnb1* mRNA levels were induced after dG treatment in WT and *Trif^−/−^* BMDMs but not in *Myd88^−/−^* and *Trif^−/−^; MyD88^−/−^* cells (Fig. 2B). mRNA levels of the interferon stimulated gene *Ifit1* were also increased upon dG treatment in a MyD88‐dependent manner, and similar results were obtained for the chemokine *Cxcl19* (Fig. 2B).

To further characterise the response induced by dG, we performed transcriptome analysis 3 h post‐dG treatment in WT, *Myd88^−/−^* and *Ifnar^−/−^* BMDMs. Five hundred and thirty‐eight genes were differentially expressed in WT cells (>2‐fold change) upon exposure to dG (Fig. 2C). The vast majority of these remained unchanged in dG‐treated *Myd88^−/−^* cells, indicating that the transcriptional response was largely dependent on MyD88. In WT cells, numerous cytokines and chemokines (such as *Il12*, *Il1b*, *Cxcl1*, and *Ccl5*), inflammatory mediators (such as *Nos2*), and feedback regulators (including *Socs3*, *Cish*, and *Dusp2*) were among the most upregulated mRNAs, some of which were validated by RT‐qPCR (Fig. 2D and E). Functional analysis revealed that genes involved in immunity and innate immune response were significantly enriched among the differentially expressed genes (Supporting Information Table S1).

It is noteworthy that *Ifit1* and *Cxcl19* mRNAs were undetectable in cells lacking the type I IFN receptor (IFNAR) at baseline, and were not induced by dG (Fig. 2B). Moreover, although WT and IFNAR‐deficient cells showed a broadly similar expression profile after dG treatment, a small subset of genes was not or only weakly induced in *Ifnar^−/−^* cells (Fig. 2C). These observations suggest that full induction of some dG‐target genes requires either sufficient baseline expression or amplification via secreted type I IFN and IFNAR signalling.

Taken together, these results showed that dG induced cytokine production and gene expression in BMDMs. These far‐reaching and rapid effects required MyD88, consistent with the idea that dG signalled through a MyD88‐dependent TLR.

To determine whether an endocytic TLR was required for dG sensing, we performed experiments in the presence of chloroquine, an inhibitor of endocytosis. Chloroquine abolished TNF and IL6 secretion upon treatment with dG and the TLR7 agonist R837 (Fig. 2F and G). We next tested if TLR7 detects dG. First, we used the oligonucleotide ODN20958 that specifically blocks TLR7. As expected, we found that TNF production triggered by R837 was blocked by ODN20958 (Fig. 2H). ODN20958 also reduced TNF secretion induced by dG (Fig. 2H). Second, *Tlr7^−/−^* BMDMs did not produce TNF upon treatment with dG or, as a control, R837 (Fig. 2I). Third, RT‐qPCR analysis showed that that *Tnf* and *Il6* mRNAs were not induced in dG‐treated *Tlr7^−/−^* BMDMs, while the response to the TLR3 agonist poly I:C was largely normal (Fig. 2J). A similar trend, although not statistically significant, was seen for *Ifit1* and *Ifnb1* mRNAs (Fig. 2J). In sum, these data demonstrate that dG was detected by TLR7.

To test whether TLR7 detects dG in other types of cells apart from BMDMs, we analysed mouse dendritic cells obtained from bone marrow cultures in the presence of Flt3 ligand. We observed TNF and IL6 secretion from bulk cultures upon treatment with dG, R837, and the TLR9 agonist CpG‐A oligonucleotide ODN1585 (Supporting Information Fig. S1A and B). The response to dG and R837 was TLR7‐dependent, while the response to the TLR9 agonist was independent of TLR7 (Supporting Information Fig. S1A and B). These cultures contain a mix of cells, including plasmacytoid dendritic cells (pDCs) 13. We identified pDCs using flow cytometry by cell surface expression of CD11c and B220 (Supporting Information Fig. S1C). We then analysed TNF production in pDCs by intracellular staining. As expected, both WT and *Tlr7^−/−^* pDCs produced TNF upon stimulation with the TLR9 agonist (Supporting Information Fig. S1C and D). Importantly, dG and R837 induced TNF in WT but not in TLR7‐deficient pDCs (Supporting Information Fig. S1C and D). These data show that not only BMDMs but also murine bone marrow derived pDCs recognise dG via TLR7.

We also asked whether dG induced a response in human cells. We stimulated fresh PBMCs from healthy volunteers with dG or R837 for 24 h. dG induced the secretion of IP10 (a chemokine also known as CXCL10) in PBMCs from four out of six analysed donors (Supporting Information Fig. S1E). We also measured induction of the interferon stimulated gene *IFI44* by RT‐qPCR and found it to be induced by dG in PBMCs from the same four donors (Supporting Information Fig. S1F). These data show that dG can induce an innate immune response in human PBMCs. Variability between donors may be related to the frequency of TLR7‐expressing cells such as pDCs, which is known to vary between individuals 14.

### TLR7 activation by dG does not require RNA

**Figure 3:**
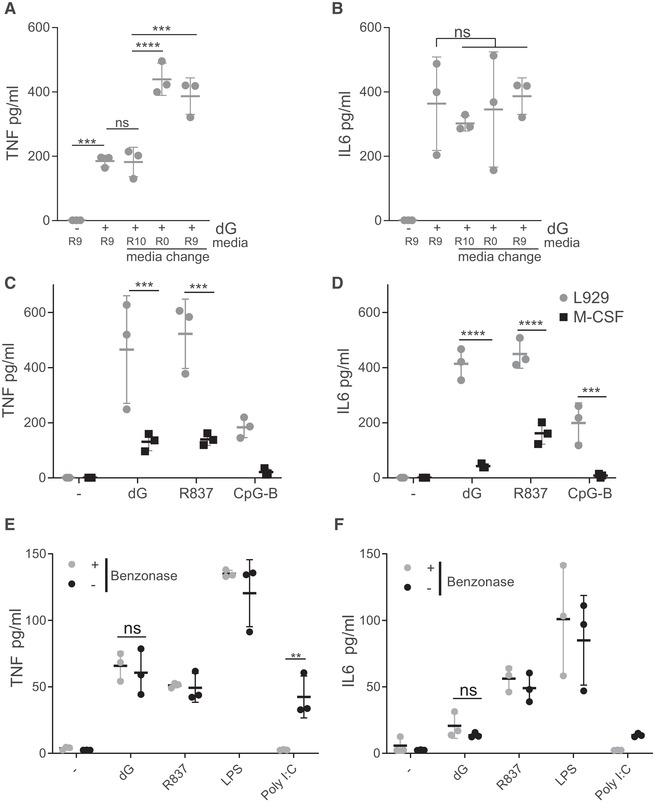
TLR7 activation by dG does not require RNA. (A, B) BMDMs were treated with 0.5 mM dG for 24 h and supernatants were analysed as in Fig. 1A and B. Before the addition of dG, media were replaced where indicated with R10 (RPMI containing 10% FCS), R0 (RPMI with no additions), or fresh R9 (RPMI containing 10% FCS and 20% L929 conditioned medium). (C, D) BMDMs were differentiated from bone marrow in R9 (L929) or in R10 with 20 ng/mL M‐CSF. BMDMs were then treated with 0.5 mM dG, 2.5 µg/mL R837, 2.5 µg/mL CpG‐B DNA, or 2.5 ng/mL LPS for 24 h. Supernatants were analysed as in Fig. 1A and B. (E, F) dG or the indicated TLR agonists were pre‐incubated with (+) or without (−) benzonase (250 U/mL) for 30 min and were then added to BMDMs (0.5 mM dG, 2.5 µg/mL R837, 2.5 ng/mL LPS, 200 ng/mL poly I:C). Supernatants were analysed after 24 h as in Fig. 1A and B. Pooled data from biological replicates (BMDM cultures originating from individual mice, *n* = 3) are shown with mean ± SD. Data are representative of three (A, C) or two (B, D–F) independent experiments. Dashed lines represent detection limits. *p*‐values determined with two‐way ANOVA are indicated. ns, *p* ≥0.05; **, *p* < 0.01; ***, *p* < 0.001; ****, *p* < 0.0001.

Previous work suggested that cytokines are not induced in WT cells treated with dG alone and that activation of TLR7 by ssRNA is enhanced by dG 4, 12. Our protocol to expand and differentiate BMDMs from bone marrow involves RPMI medium supplemented with fetal calf serum (FCS) and conditioned medium from L929 cells (designated R9). We replaced the culture media before dG addition with RPMI medium containing only FCS (R10), RPMI medium without supplements (R0), or fresh R9 medium. In all of these conditions, TNF and IL6 were produced upon treatment with dG (Fig. 3A and B). This suggested that neither FCS nor L929 conditioned medium contained RNAs that activated TLR7 in conjunction with dG, and that immunostimulatory RNAs did not accumulate over time during the differentiation process in R9. To compare differentiation protocols, we grew BMDMs either in R9 (used in all our BMDM experiments) or in R10 supplemented with 20 ng/mL of recombinant M‐CSF. BMDMs differentiated with M‐CSF produced TNF and IL6 upon dG treatment, although at lower levels compared to cells grown in R9 (Fig. 3C and D). This was not specific to dG since R837 and CpG‐B were also less potent in BMDMs differentiated in R10 and M‐CSF (Fig. 3C and D). This suggested that the differentiation protocol did not qualitatively determine the response to dG in the absence of RNA. To test if dG was contaminated with RNA, we added benzonase — a nuclease that degrades DNA and RNA — to dG or control agonists prior to stimulation of BMDMs. As expected, benzonase treatment did not diminish the activity of R837 and LPS to induce TNF and IL6 (Fig. 3E and F). However, the RNA poly I:C failed to induce these responses after benzonase treatment, showing that the enzyme was active in our setting (Fig. 3E and F). Importantly, pre‐treatment with benzonase did not reduce TNF and IL6 secretion upon dG stimulation in BMDMs (Fig. 3E and F). Taken together, these observations suggest that TLR7 activation by dG did not require the presence of RNA.

## Concluding remarks

Here, we show that TLR7 detects dG, expanding the spectrum of agonists for this PRR. Interestingly, we find that concomitant provision of ssRNA is not required for activation of TLR7 by dG. This mimics the situation with the synthetic TLR7 agonist R848 10. In the future, it will therefore be interesting to determine the structure of TLR7 in complex with dG. In contrast to our observations, earlier studies reported that dG does not activate TLR7 in WT cells 4, 12. We speculate that these differences may be due to assay sensitivity and the type and/or activation status of the cells used; indeed, Shibata et al. found that dG treatment induces cytokines in hyperresponsive *Unc93b1* mutated cells 12.

Are there physiological situations in which dG may act as PAMP or danger‐associated molecular pattern for TLR7? The enzyme purine nucleoside phosphorylase (PNP) degrades dG. Patients suffering from PNP deficiency, a rare immunodeficiency, have elevated plasma dG concentrations 15. Moreover, a *PNP* polymorphism was identified in a systemic‐lupus erythematosus patient and linked to enhanced type I IFN induction 16. We speculate that dG sensing by TLR7 exacerbates these phenotypes. Finally, PNP inhibitors such as forodesine are developed as treatments for cancer and induce increased dG levels in blood 17. If and how dG sensing by TLR7 influences the efficacy of these compounds should be considered in future studies.

## Material and methods

### Mice

Mice were on the C57BL/6 background. *Samhd1^−/−^* mice were described previously 18. Bone marrow samples from *Myd88^−/−^, Trif^−/−^*, and *Trif^−/−^; Myd88^−/−^* mice were a gift from K. Maloy (mice originally from S. Akira). *Ifnar^−/−^* and *Tlr7^−/−^* mice and bone marrow from *Tlr4^−/−^* mice were gifts from C. Reis e Sousa (mice originally from M. Aguet and S. Akira, respectively). STING‐deficient mice (*Mpys^−/−^*) were from J. Cambier. This work was performed in accordance with the UK Animals (Scientific Procedures) Act 1986 and institutional guidelines for animal care and was approved by a project license granted by the UK Home Office (PPL No. PC041D0AB) and the Institutional Animal Ethics Committee Review Board at the University of Oxford.

### Cell culture

Bone marrow cells were isolated by standard protocols. To obtain BMDMs, cells were grown in petri dishes for 7 days in R9 medium (Roswell Park Memorial Institute 1640 (RPMI) medium, 10% v/v heat‐inactivated FCS, 100 U/mL penicillin and 100 µg/mL streptomycin, 2 mM L‐Glutamine, 20% v/v L929 conditioned medium). Where indicated, BMDMs were differentiated using 20 ng/mL of M‐CSF instead of L929 conditioned medium. BMDMs were seeded on day 7 in 96‐well plates (20 000–40 000 cells/well) and treated the next day. To obtain pDC‐containing cultures, bone marrow cells were grown in 200 ng/mL Flt3 ligand instead of L929 conditioned medium. After 8 days, 2 × 10^6^ cells were seeded in 200 µL in 96 well plates and treated.

### PBMCs

PBMCs were isolated from fresh blood from healthy volunteers using standard methodology. 10^6^ cells were seeded in 500 µL in 48‐well plates and treated for 24 h. This work was carried out in accordance with the EU Directive 2004/23/EC and the UK Human Tissue Act 2004 (licence number 12433).

### ELISA

TNF and IL‐6 were quantified in technical duplicate by ELISA according to manufacturer's instruction. 96‐well plates were used with 50 µL sample per well.

### Flow cytometry

Flt3 ligand cultures were treated with 10 µg/mL brefeldin A during the final 4 h of stimulation and were prepared for FACS analysis as described before 19. We adhered to EJI guidelines for FACS 20. Cells were analysed on an Attune Flow Cytometer (Thermofisher Scientific).

### RT‐qPCR

RT‐qPCR was performed as described previously 18.

### Microarray analysis

RNA was extracted using Qiagen RNeasy mini kit. Samples were analysed using the mouse Clariom™ S Assay HT. Results were normalised and genes with <2‐fold changes between treated and non‐treated groups were excluded as well as those with FDR > 0.05. Morpheus (https://software.broadinstitute.org/morpheus) was used to generate the heatmap. DAVID (v6.8) was used to perform gene annotation clustering. Significant differentially expressed genes were calculated with R using the limma package 21 comparing dG vs. untreated.

## Conflict of interest

The authors declare no financial or commercial conflict of interest.

**Abbreviations**
BMDM: bone‐marrow derived macrophages
dG: deoxyguanosine
dN: deoxyribonucleoside
FCS: fetal calf serum
pDC: plasmacytoid dendritic cells
PNP: purine nucleoside phosphorylase
ssRNA: single strand RNA
TIR: Toll/interleukin‐1 receptor homology

## Supporting information

Figure S1. dG induces innate immune responses in murine pDCs and human PBMCs.
Table S1. Functional analysis of dG‐regulated genes.
Table S2. Reagents.
