# Supplementary material for: Deoxyguanosine is a TLR7 agonist
Source: Eur J Immunol. 2019 Nov 14;50(1):56–62. doi: 10.1002/eji.201948151 (PMC6972671; doi:10.1002/eji.201948151)
Supplement: Supplementary file 1 — Figure S1. dG induces innate immune responses in murine pDCs and human PBMCs. Table S1. Functional analysis of dG‐regulated genes. Table S2. Reagents. [file EJI-50-56-s001.docx]

Supporting information

**Deoxyguanosine is a TLR7 agonist**

Tamara Davenne^1,2^, Anne Bridgeman^1^, Rachel E. Rigby^1^ and Jan Rehwinkel^1,^*

^1^Medical Research Council Human Immunology Unit, Medical Research Council Weatherall Institute of Molecular Medicine, Radcliffe Department of Medicine, University of Oxford, Oxford OX3 9DS, UK.

^2^current address: Laboratory for Disease Mechanisms in Cancer, Department of Oncology, KU Leuven and Leuven Cancer Institute (LKI), Herestraat 49, 3000 Leuven, Belgium.

^*^ Correspondence: [jan.rehwinkel@imm.ox.ac.uk](mailto:jan.rehwinkel@imm.ox.ac.uk)

**Figure S1. dG induces innate immune responses in murine pDCs and human PBMCs.**

(**A**-**D**) Ftl3 ligand bone marrow derived dendritic cell cultures of the indicated genotypes were treated with 0.5 mM dG, 2.5 μg/ml R837 or 2.5 μg/ml CpG-A DNA for 24 hours. (A,B) Supernatants were analysed as in Fig. 1A,B. (C,D) pDCs were analysed by flow cytometry. The gating strategy for B220^+^CD11c^+^ pDCs (top) and representative FACS plots for intracellular TNF staining in pDCs (bottom) are shown in (C). Panel (D) shows TNF^+^ pDCs. Pooled data from biological replicates (BMDM cultures originating from individual mice, n=3) are shown with mean ± SD. Data are representative of two independent experiments.

(**E**,**F**) Fresh human PBMCs were treated with 0.5 mM dG or 2.5 μg/ml R837 for 24 hours. (F) Supernatants were tested for IP10 by ELISA. (G) RNA was extracted and *IFI44* expression was analysed by RT-qPCR. Data are relative to 18S rRNA. Pooled data from two independent experiments, each with three PBMC donors, and are shown with mean ± SD. Colours represent PBMC donors and data points are the mean of technical triplicates (F) or duplicates (G).

P-values determined with two-way ANOVA are indicated. ns, p≥0.05; *, p<0.05; **, p<0.01; ***, p<0.001; ****, p<0.0001.

Table S1. Functional analysis of dG-regulated genes.

Gene annotation clustering was performed with DAVID on the list of 538 genes differentially expressed in WT cells post dG treatment. Enrichment scores, number of genes and p-values are shown.


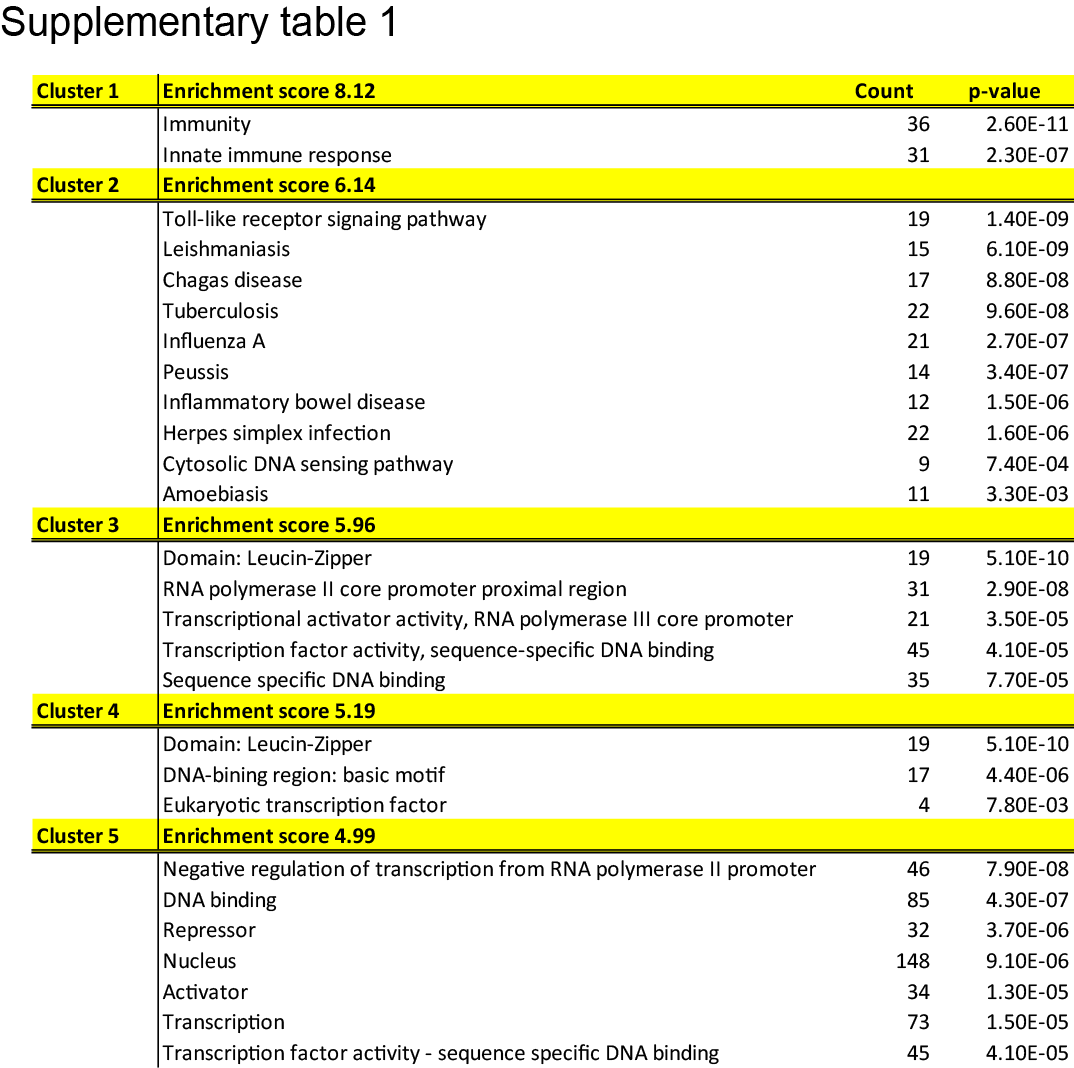


**Table S2. Reagents.**

| **Reagent** | **Supplier** | **Cat. Nb.** |
| --- | --- | --- |
| IL-6 Mouse Uncoated ELISA Kit | Life Technologies Ltd | 88-7064-88 |
| TNF alpha Mouse Uncoated ELISA Kit | Life Technologies Ltd | 88-7324-88 |
| Human CXCL10/IP-10 DuoSet ELISA | R&D | DY266-05 |
| Imiquimod (R837) | Invivogen | tlrl-imqs |
| Benzonase | Merck Chemicals Ltd | 70664-3 |
| ODN 2088 Control (ODN 20958) | Miltenyi Biotec Ltd | 130-105-821 |
| M-CSF Recombinant Mouse Protein | Life Technologies Ltd | PMC2044 |
| LPS | Sigma | L7770 |
| Flt3 ligand recombinant mouse protein | R&D | 427-FL-005 |
| Brefeldin A | Sigma | B6542 |
| LIVE/DEAD Fixable Violet Dead Cell Stain Kit | Life Technologies Ltd | L34955 |
| APC/Cy7 anti-mouse/human CD45R/B220 | Biolegend | 103224 |
| PerCP/Cy5.5 anti-mouse CD11c | Biolegend | 117328 |
| PE anti-mouse TNF | Biolegend | 506306 |
| 2' deoxyguanosine | MedChemexpress LLC | HY-17563 |
| 2' deoxycytidine | MedChemexpress LLC | HY-17564 |
| 2' deoxythymidine (thymidine) | Sigma | T1895-1G |
| 2' deoxyadenosine | Sigma | D8668-1G |
| *Socs3* mouse taqman probe | Life Technologies Ltd | Mm00545913_s1 |
| *Tpbg* mouse taqman probe | Life Technologies Ltd | Mm00495741_s1 |
| *Nos2* mouse taqman probe 75reaction | Life Technologies Ltd | Mm00440502_m1 |
| *Cish* mouse taqman probe | Life Technologies Ltd | Mm01230623_g1 |
| *Il12b* mouse taqman probe | Life Technologies Ltd | Mm01288989_m1 |
| *Ptgs2* mouse taqman probe | Life Technologies Ltd | Mm00478374_m1 |
| *Tnf* mouse taqman probe | Life Technologies Ltd | Mm00492606_m1 |
| *Ifit1* mouse taqman probe | Life Technologies Ltd | Mm00515153_m1 |
| *Ifnb1* mouse taqman probe | Life Technologies Ltd | Mm00439546_s1 |
| *Il6* mouse taqman probe | Life Technologies Ltd | Mm00446191_m1 |
| *IFI44* human taqman probe | Life Technologies Ltd | Hs00951349_m1 |
| CpG-B DNA (ODN 2006) | Hycult Biotech | HC4039 |
| CpG-A DNA (ODN1585) | Invivogen | tlrl-1585 |
| poly(I:C) HMW | Invivogen | tlrl-pic |
| Corning 96-well plate half-area high binding | Scientific laboratory supplies Ltd | 3690 |
